# Electrocardiogram features of premature ventricular contractions/ventricular tachycardia originating from the left ventricular outflow tract and the treatment outcome of radiofrequency catheter ablation

**DOI:** 10.1186/1471-2261-12-112

**Published:** 2012-11-27

**Authors:** Bei Ge, Kang-Ting Ji, Hai-Ge Ye, Jia Li, Yue-Chun Li, Ri-Peng Yin, Jia-Feng Lin

**Affiliations:** 1 Department of Cardiology, Second Affiliated Hospital of Wenzhou Medical College, 109 Xueyuan Road, Wenzhou, Zhejiang, China; 2 Department of Hematology, First Affiliated Hospital of Wenzhou Medical College, Wenzhou, China

**Keywords:** Electrophysiology, Ventricular arrhythmia, Left ventricular outflow, Catheter ablation, Radiofrequency current

## Abstract

**Background:**

Radiofrequency catheter ablation (RFCA) has been used for the ablation of premature ventricular contractions (PVCs) or ventricular tachycardia (VT). To date, the mapping and catheter ablation of the arrhythmias originating from the left ventricular outflow tract (LVOT) has not been specified. This study investigates the electrocardiogram (ECG) feature of PVCs or VT originating from the LVOT. Moreover, the treatment outcome of RFCA is analyzed.

**Methods:**

Mapping and ablation were performed on the supravalvular or subvalvular aorta in 52 cases with PVCs/VT originating from the LVOT. The data were compared with those from 104 patients with PVCs/VT originating from the right ventricular outflow tract (RVOT). A differential procedure was prepared based on the comparison of the ECG features of PVCs/VT originating from the RVOT, LVOT, and their different parts.

**Results:**

Among 52 cases with PVCs originating from the LVOT, 47 were successfully treated by RFCA, with a success rate of 90.38%. Several differences among the 12-lead ECG features were observed from the RVOT and LVOT in the left and right coronary sinus groups, as well as under the left coronary sinus group (left fibrous trigone): (1) If the precordial leads transition <V3 plus the precordial leads transitional index >0 are considered as the diagnostic parameters of PVCs/VT originating from the LVOT, then the sensitivity, specificity, as well as positive and negative predictive values are 94.12%, 93.00%, 87.27%, and 96.88%, respectively; (2) The analysis of different subgroups of the LVOT are as follows: (a) A mainly positive wave of r or m pattern was recorded in the lead I in 72.73% of patients in the right coronary sinus group, versus 12.90% of patients in the left coronary sinus group, and 0% in the under left coronary sinus group. (b) All patients in the right coronary sinus group presented waves of R_II_>R_III_ and QS_aVR_>QS_aVL_, whereas most patients in the other two groups showed waves of R_III_>R_II_ and QS_aVL_>QS_aVR_. (c) Most patients in the under left coronary sinus group in lead V1 had a mainly positive wave (R) (77.78%), whereas those in the right (81.82%) and left (62.50%) coronary sinus groups had mainly negative waves (rS).

**Conclusions:**

RFCA is a safe and effective curative therapy for PVCs/VT originating from the LVOT. The 12-lead ECG features of the LVOT from different origins exhibit certain distinctions.

## Background

Most cases of idiopathic ventricular tachycardia (VT) and premature ventricular contractions (PVCs) originate from the right ventricular outflow tract (RVOT) and the left ventricular postmedian septum. Only a few VT and PVCs originate from the left ventricular outflow tract (LVOT) and the vicinity of the atrioventricular wreath. In the present study, radiofrequency catheter ablation (RFCA) was performed on 52 cases of idiopathic PVCs/VT originating from different parts of the LVOT. The electrocardiogram (ECG) features of these cases were investigated and compared with those of 104 cases of idiopathic PVCs/VT originating from the RVOT. A differential procedure was prepared based on the comparison and analysis.

## Methods

### Study population

Between July 2006 and December 2011, 553 patients with idiopathic PVCs/VT were treated with RFCA in our hospital. Among the cases, 52 originated from the LVOT (9.40%), including 16 males and 36 females, aged 60.58 ±14.81 (19–79), in the course of 3.08 ± 2.36 (0.5–14) years. All patients had different degrees of palpitations and tightness of the chest. Administration of two to three types of antiarrhythmic drugs was ineffective, and this approach significantly affected their qualities of life. Routine examination of biochemistry, chest X-rays, echocardiography, and other tests yielded no structural heart disease in the 52 cases (except for mild enlargement of the left ventricular in four cases: the left ventricular end-diastolic internal diameter was between 57 and 62 mm, and their re-examination showed full recovery when the PVCs/VT were treated by ablation for half a year). The mean left ventricular end-diastolic internal diameter was 47.82 ± 4.53 mm (range 42–62). All patients were examined via dynamic cardiogram to monitor the quantity and onset feature of 24 h PVCs/VT. This test showed that the sum of PVCs/VT was 20,716 ± 11,052 (11,079–48,972) beats/24 h. Among the cases, 44 were simple PVCs with clinical manifestations, 7 were PVCs with paroxysmal VT, 1 was sustained VT, and 2 showed a slightly reduced ejection fraction of the left ventricular (52% and 49%). All patients were referred for ablation. The ECG features of the cases originating from the LVOT and RVOT were compared. A total of 104 patients with PVCs/VT originating from the RVOT in the same period were randomly selected for the controlled analysis. These patients were successfully treated with RFCA. Among these RVOT cases, 36 were males and 68 were females, aged 48.33 ± 15.7 (15–78), with 11 suffering from VT and 93 from PVCs.

### Ethical approval

Ethical approval was obtained from the Ethics Committee of the Second Affiliated Hospital of Wenzhou Medical College. All patients signed informed consent before participation in this study.

### ECG examination and measurement

Examination through 12-lead synchronous surface ECG showed no T wave inversion of precordial leads and epsilon wave in all patients. Observation of the QRS complex morphology in every lead at the onset of PVCs/VT obtained the following measurements: height of the R or r wave as well as the depth of the S or s wave in every lead; calculation of the R wave amplitude in V1–V2 leads (height of the R wave/depth of the S wave) and the R wave duration index [1] (measurement of R/QRS wave duration during cardiac electrophysiology examination; paper speed 100 mm/s); as well as consideration of the transitional region and calculation of the transitional index in precordial leads during PVCs/VT and sinus rhythm (transitional index in precordial leads=transitional region in precordial leads during sinus rhythm-transitional region in precordial leads during PVCs/VT) [2]. This study focuses on the relatively tall amplitude wave (>0.5 mV) of Q, R, and S, as well as the relative low amplitude wave (≤0.5 mV) of q, r, and s.

### Intracardiac electrophysiologic examination and radiofrequency ablation treatment

All patients discontinued the use of antiarrhythmic drugs for at least five half lives before operation. A Judkins L3.5 or R3.0 angiographic catheter was routinely placed at the ostium of the left or right coronary artery from the radial artery. If necessary, a 2-pole or 4-pole mapping catheter and a 10-pole coronary sinus mapping catheter could be placed at the right ventricular apex or at the right atrium and the coronary sinus. In addition, procedural and non-procedural stimuli, as well as mapping, were performed in basal conditions. Isoproterenol was administered intravenously. Furthermore, a 7F arterial sheath was placed at the right femoral artery. Mapping and ablation were performed via intubation of a 4-pole ablation catheter into the LVOT (above or under the semilunar valve) directly through the sheath, according to the primary location showed by the surface ECG before operation.

Mapping and ablation of spontaneous PVCs or of PVCs induced by isoproterenol and pace stimulus mainly relied on the activation sequence mapping and were supplemented by pace mapping. The ventricle was paced at a slow rate of 120 to 140 per minute, according to the coupling interval of the PVC. During pacing, 12-lead ECG and QRS waves of spontaneous PVCs were the same in at least 11 leads. Alternatively, the ablation target site considered as the QRS wave on the endocardial electrogram of the activation sequence mapping was 25 ms ahead that of PVCs, which was treated by ablation when the PVCs became frequent, following mapping and location through electrophysiologic examination. The location link between the target site and the coronary requires identification through coronary angiography before ablation in all patients. Ablation should cease upon noticing the catheter transposition in continuing fluoroscopy of the ablation catheter. Repeat coronary angiography could be performed to observe the status of the blood supply after a successful ablation. If the ideal target site is not mapped in the LVOT, the RVOT should be mapped. If no ideal target site is observed, mapping and ablation could finally be guided by the mapping ablation catheter (quadripolar irrigated-tip catheter with a 4-mm distal electrode) from the coronary sinus to the distal great cardiac vein (if necessary, the mapping ablation catheter could be guided by coronary venography). A ablation catheter of temperature-controlled ablation was generally selected. The preset temperature was 52°C to 55°C, the preset energy was 30 W to 50 W, and impedance was 80 Ω to 140 Ω. However, saline-irrigated ablation catheter should be the priority when the mapping ablation catheter is guided from the coronary sinus to the distal great cardiac vein. In this case, the preset temperature was 43°C, the preset energy was 30 W to 35 W, and the saline-irrigated velocity must be adjusted to its maximum (60 ml/min). Ablation was attempted for 10 s after the temperature reached 50°C (temperature of the saline-irrigated catheter was 43°C). The effective target site was defined as a point when PVCs disappeared within 10 s after ablation, when VT terminated, or when frequent PVCs of the same form appeared with spontaneous PVCs and paroxysmal VT during ablation, then shortly disappeared. After continuing ablation for 60 s to 180 s at the effective target site and supplementing point ablation around the site, monitoring continued for 30 min. Terminating the ablation was determined when PVCs disappeared and when the initial induced PVCs/VT methods, such as power stimulus and intravenous administration of isopropylarterenol, were not effective. Re-mapping of target site is necessary if PVCs do not disappear after ablation for 10 s or if VT could not be terminated.

### Follow-up methods

(1) Routine ECG monitoring for 48 hours after ablation operation; (2) patient re-examination once in three months after operation using echocardiogram plus dynamic ECG to estimate the long-term effect; during this period, all administration of anti-arrhythmia drugs should cease; (3) further and timely consultation for any special condition alterations during outpatient follow-up; (4) archival of each patient. Patients were followed up by telephone in 6, 12, and 18 months after the ablation procedure. ECG and 24-hour ECG monitoring were performed whenever the patient had symptoms suggestive of recurrence of PVCs/VT.

### Definition of successful ablation

The standard of instant success are as follows [3]: when PVCs disappear or when sporadic PVCs (≤1 beats/min)/VT cannot be induced after radiofrequency ablation; and when close observation for 30 min after operation reveals a reduction of the total number of PVCs to less than10 (shape is completely similar to monomorphous PVCs before operation). The standard of long-term success is when dynamic ECG monitoring for a full day within three months after operation show that PVCs disappeared, or when total number of PVCs decreased by over 75%. Moreover, long-term success is achieved when the VT does not recur and the uncomfortable symptoms are remarkably improved.

### Statistical analysis

Measurement data are described as Mean ± Standard deviation ($\bar{\text{x}}\pm \text{s}$). Independent samples were applied for comparison among groups using the *t*-test, ANOVA analysis, and the Q test. Enumeration data are described as case numbers and percentages. The probability could be directly measured among groups through X-squared tests or Fisher’s exact test. P < 0.05 is considered as statistically significant.

## Results

### Mapping and ablation results of PVCs/VT originating from the LVOT

All patients were treated with mapping and ablation applied through temperature controlled catheter ablation. Successful ablation was achieved for 47 out of 52 patients (90.38%). Simple activation sequence mapping was performed on 34 cases, among which 30 succeeded. Activation sequence combined with pace mapping were performed on 18 cases, among which 17 succeeded (in these 17 cases, the QRS complex of pacing from the effective target site and spontaneous PVCs in 12-lead surface ECG were common in at least 11 leads). The average operation time was 73.46 ± 26.72 (35.00–120.00) min beginning from puncturing to pulling out the sheath. The average X-ray exposure time was 10.13 ± 4.12 (3.10–21.3) min. The average ablation time was 258.05 ± 73.68 (90–360) s. The starting point of the ventricular potential of the effective target site was 35.83 ± 6.38 (26–57) ms earlier than the onset of the QRS wave on PVCs of the surface ECG in 47 patients with successful ablation. Among these cases, six showed the peak fractionated potential in the ventricular potential of the effective target site (at the terminal ventricular potential during sinus rhythm and reversed during PVCs), and two showed mid-diastole potential. The effective target site during ablation manifested that PVCs in 39 cases disappeared within 10 s after ablation and increased frequent PVCs with the same shape as spontaneous PVCs or paroxysmal VT, which shortly disappeared in 8 cases. According to the effective target site or the activation mapping of the earliest activation with X-ray image localization, the patients were divided into three groups: (1) 32 cases in the left coronary sinus group, of which 28 had successful ablation (87.50%); (2) All 11 cases in the right coronary sinus group had successful ablation (100.00%); (3) 9 cases in the left fibrous trigone under the left coronary sinus group (abbreviated as under left coronary sinus group), among which 8 cases had successful ablation (88.89%). In five failed cases, the left main coronary artery mapping in four cases showed that the earliest ventricular potential time was 25 ms to 33 ms earlier than the onset of the QRS wave on the PVCs of surface ECG. Ablation was then ceased. In the other case, under front left coronary sinus (about 0.5 cm from the left coronary sinus) mapping showed that the ventricular potential time was up to 34 ms earlier than the onset of the QRS wave on the PVCs of surface ECG, and repetitive ablation was ineffective. All patients were monitored by ECG for three successive days after operation and were re-examined by dynamic ECG within three months after operation. The mean duration for follow up lasted 12.9 ± 10.4 (3–34) months. Two cases relapsed. Tables 1 and 2 compare the general clinical status and the results of mapping and ablation in three groups, respectively.

**Table 1:** Comparison of general clinical conditions among the three groups

**Group**	**Sex (male%)**	**Age (years)**	**Duration (years)**	**PVCs total number (number/24 h)**	**With hypertension**	**With diabetes**	**With left ventricular enlargement**	NSVT (SVT)*	**PVCs**
Left coronary sinus (n = 32)	9(28.13)	60.08 ± 13.82	3.06 ± 2.37	20378 ± 9825	12(37.50)	3(9.38)	2(6.25)	4(1)	28(87.50)
Right coronary sinus (n = 11)	4(36.36)	62.09 ± 14.27	2.96 ± 2.52	21848 ± 11741	5(45.45)	2(18.18)	1(11.11)	2(0)	9(81.82)
Under left coronary sinus (n = 9)	3(33.33)	60.52 ± 17.72	3.28 ± 2.43	20537 ± 11374	3(33.33)	1(11.11)	1(11.11)	2(0)	8(77.78)

*Abbreviation: *NSVT* non-sustained ventricular tachycardia, *SVT* sustained ventricular tachycardia.

**Table 2:** Comparison of the results of mapping and ablation among the three groups

**Group**	**Activation of mapping V-QRS time (ms)**	**Mapping cases with effective target site pace (%)**	**Ablation times**	**Ablation time (s)**	**Operation time (min)**	**Exposure time of X-rays (min)**	**Effective ablation cases (%)**
Left coronary sinus (n = 32)	35.62 ± 4.17	3(9.38)	2.79 ± 1.42	249.23 ± 71.68	73.01 ± 26.18	10.11 ± 4.23	28(87.50)
Right coronary sinus (n = 11)	35.60 ± 3.92	8(72.73)*	2.85 ± 1.58	268.81 ± 74.36	75.23 ± 27.84	10.57 ± 4.11	11(100.00)
Under left coronary sinus (n = 9)	35.75 ± 5.44	8(88.89)^△^	1.67 ± 0.56^△☆☆^	276.27 ± 56.41	71.89 ± 34.48	9.67 ± 3.88	8(88.89)

Note: Comparison of the right coronary sinus group with the left coronary sinus group, *P < 0.01; Comparison of the under left coronary sinus group with the left coronary sinus group, △ < 0.01; Comparison of the under left coronary sinus group with the right coronary sinus group, ☆☆p < 0.05.

Table 1 shows that no statistical difference in the general clinical status among the three groups (p > 0.05). Table 2 indicates no significant difference in the V-QRS time of activation mapping, operation time, X-ray exposure time, ablation time, and successful rate of ablation among the three groups (p > 0.05). The number of cases with pace mapping of the effective target site in the left coronary sinus group was significantly less than that in the right coronary sinus and the under left coronary groups (both with p < 0.01). However, the number of ablations in the under left coronary sinus group was significantly less than that in the left coronary sinus and the right coronary sinus groups (p < 0.01, p < 0.05, respectively).

### Comparison of the ECG features of PVCs/VT originating from the RVOT and LVOT

The PVCs/VT in leads II, III, aVF, and V5–V6 presented single-way R wave, regardless of origin. Table 3 compares the 12-lead ECG of the two groups and every subgroup of PVCs/VT that originated from the LVOT.

**Table 3:** **Comparison of the features of QRS complex on surface 12-lead ECG in two groups (cases**%**)**

**Group**	**I QRS shape**				**R**_**II**_ **> R**_**III**_		**QS**_**aVR**_ **> QS**_**aVL**_		**R wave descending notch in II, III, aVF leads**	
	**Rs**	**rs or rS**	**R, r, or m**	**qs or qr**						
RVOT (n = 104)	4(3.85)	37(35.58)	45(43.27)	18(17.31)	59(56.73)		59(56.73)		16(15.38)	
LVOT (n = 52)	6(11.54)	33(63.46) #	12(23.08)##	1(1.92) ##	19(36.54) ##		19(36.54) ##		8(15.38)	
Left coronary sinus (n = 32)	1(3.13)	26(81.25)	4(12.50)	1(3.13)	6(18.75)		6(18.75)		0☆	
Right coronary sinus (n = 11)	2(18.18)	1(9.09) *△	8(72.73) *△	0	11(100.00) *△		11(100.00) *△		0*	
Under left coronary sinus (n = 9)	3(33.33)	6(66.67)	0	0	2(22.22)		2(22.22)		8(88.89)	
V1 QRS complex shape			V2 QRS complex shape			Precordial leads transition				
rS	R	RS or Rs	rS	R	RS orRs	≤V1	≤V2	<V3	≥V3	Transitional index >0
99(95.19)	0	5(4.81)	94(91.00)	2(1.92)	8(7.69)	2(1.92)	7(6.73)	17(16.35)	78(75.00)	6(5.77)
29(55.77) #	8(15.38)#	15(28.85)#	12(23.08)#	16(30.77)#	24(46.15)#	22(42.31)#	15(28.85)#	12(23.08)	3(5.77)#	49(94.23)#
20(62.50) ☆	1(3.13) ☆	11(34.38)	5(15.63)	7 (21.88)	20(62.50) ☆	11(34.38)	14(44.16)	7(22.58)	/	30(93.75)
9(81.82) △	0 △	2(22.22)	7(63.64) **△	0 △	4(36.36)	2(22.22) △	1(11.11) *△	5(45.45) **△	3(27.27)	10(90.91)
0	7(77.78)	2(22.22)	0	9(100.00)	0	9(100.00)	0	0	/	9(100.00)

Note: Precordial leads transition index [8] = Precordial leads transitional region at sinus rhythm - Precordial leads transitional region at premature ventricular contractions; Comparison of RVOT and LVOT groups #p < 0.01, ## p < 0.05; Comparison of right coronary sinus group with left coronary sinus group *p < 0.01,** p < 0.05; Comparison of right coronary sinus group with under left coronary sinus group △p < 0.01,△△p < 0.05; Comparison of left coronary sinus group with under left coronary sinus group ☆p < 0.01.

The main difference between the RVOT and LVOT groups was in their right precordial leads. The RVOT group presented mainly an rS type of S wave in 95.19% of lead V1, whereas the LVOT group presented mainly R, RS, or Rs types of R wave in 43.14% of lead V1. The former had only 6.73% of precordial leads transitional area ≤lead V2, whereas the latter had up to 71.15% (p < 0.01). In addition, only six cases had precordial leads transitional index >0 in the RVOT group (5.77%), whereas 49 cases were observed in the LVOT group (94.23%). Significant differences between the two groups (p < 0.01) were observed. Analysis of the LVOT subgroups demonstrated the following: (1) Most patients in the right coronary sinus group presented mainly r or m type of positive wave in the lead I (72.73%) verus the left coronary sinus group (12.50%) and the under left coronary sinus group (0%) (both with p < 0.01). However, most of the patients in the latter two groups (left coronary sinus and under left coronary sinus) had rs or rS type, accounting for 81.25% and 66.67%, respectively, which were significantly more than 9.09% in the right coronary sinus group (both with p < 0.01). (2) Although all patients in the three groups had R type in leads II, III, aVF, and V4–V6, and then QS type in leads aVR and aVL, differences were observed in the distribution of the height of the R wave and the depth of the QS wave. All patients in the right coronary sinus group had R_II_>R_III_, QS_aVR_>QS_aVL_, whereas most patients in the other two groups had R_III_>R_II_, QS_aVL_>QS_aVR_ (both with p < 0.01). (3) Differences were likewise observed in the shape of the QRS complex in precordial leads among the three groups. Most patients in the under left coronary sinus group presented mainly R type of positive waves in lead V1 (77.78%), whereas most patients in the right coronary sinus (81.82%) and the left coronary sinus (62.50%) groups presented rS type of negative wave. (4) All patients in the under left coronary sinus group had transition of precordial leads prior to V1, whereas those in the left and right coronary sinus groups were mainly in the V2–V3 leads. (5) Most patients in the under left coronary sinus group had a notch on the descending branch of the R wave in inferior leads (88.89%), whereas none were observed in the other two groups. The analysis considered the following: the transition of precordial leads <V3 and transitional index of precordial leads >0 as the diagnostic index of PVCs/VT that originated from the LVOT; the r or m type, R_II_>R_III_ and QS_aVR_>QS_aVL_ in the lead I as the diagnostic index of PVCs/VT that originated from the right coronary sinus; and the one-way R type in V1–V6 leads and the notch on the descending branch of the R wave in inferior leads as the diagnostic index for PVCs/VT that originated from the under left coronary sinus. The sensitivity, specificity, as well as the positive and negative predictive values are presented in Table 4.

**Table 4:** Diagnostic value of different indexes for PVCs/VT originating from different sites of LVOT

**Diagnosis index**	**Sensitivity**	**Specificity**	**Positive predictive value**	**Negative predictive value**
Precordial leads transition < V3+Precordial leads transition index >0 diagnosis of the origin of LVOT	49/52(94.23)	95/104(91.35)	49/58(84.48)	95/98(96.94)
I lead present as type r or m to diagnose the origin of the right coronary sinus	6/9(66.67)	37/41(90.24)	6/10(60.00)	37/40(92.50)
R_II_ > R_III_ to diagnose the origin of the right coronary sinus	11/11(100.00)	33/41(80.49)	11/19(57.89)	33/33(100.00)
QS_aVR_ > QS_aVL_ to diagnosis the origin of the right coronary sinus	11/11(100.00)	33/41(80.49)	11/19(57.89)	33/33(100.00)
All V1–V6 present as one-way R wave to diagnose the origin of the under left coronary sinus	8/9(88.89)	43/43(100.00)	8/8(100.00)	43/44(97.73)
Inferior leads descending a notch to diagnose the origin of the under left coronary sinus	8/9(88.89)	43/43(100.00)	8/8(100.00)	43/44(97.73)

### Results of ECG feature and ablation study in typical cases

**Figure 1:**
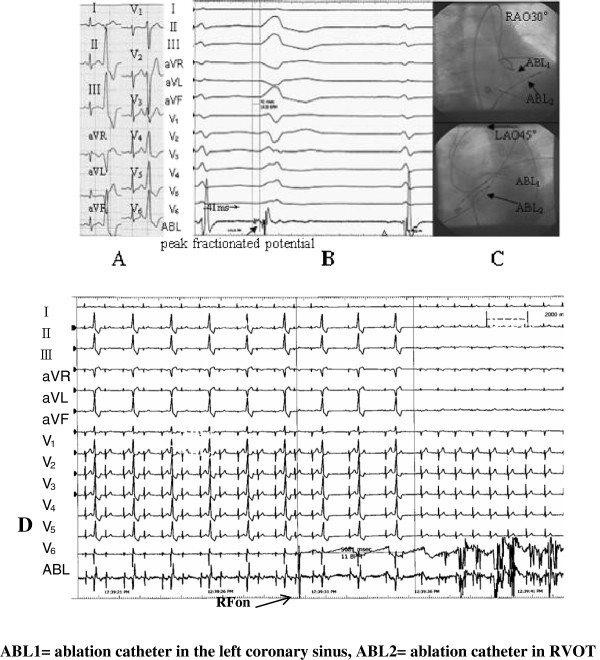
Surface 12-lead ECG feature of PVC that originated from the left coronary sinus, and mapping of the activation sequence of the effective target site, the X-ray image, and the effective feature of response during ablation.

**Figure 2:**
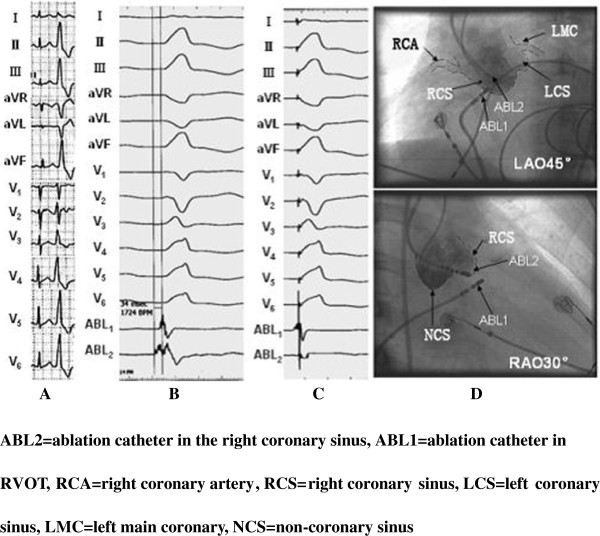
Feature of PVC on the surface 12-lead ECG that originated from the right coronary sinus, and the activation sequence of the effective target site, pace mapping, and the feature of X-ray images.

**Figure 3:**
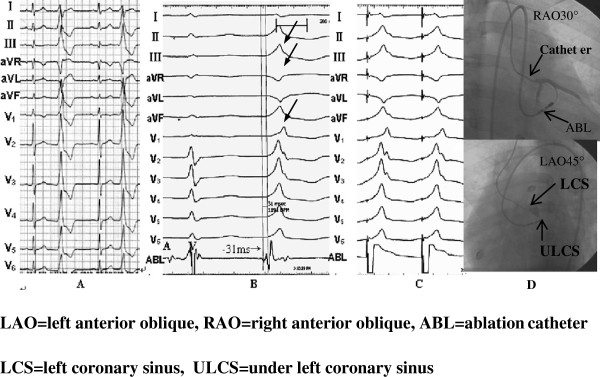
Surface 12-lead ECG feature of the PVC that originated from the under left coronary sinus; effective target site pacing, activation sequence mapping, and the feature of X-ray images.

**Figure 4:**
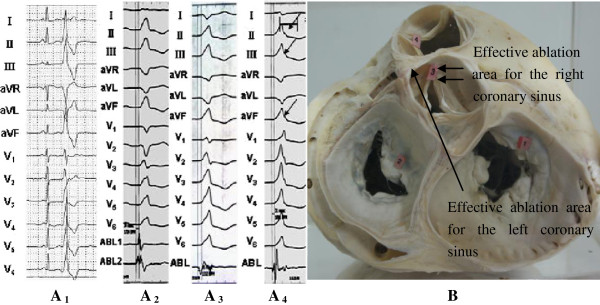
Schematic diagram of the surface 12-lead ECG characteristics for the three groups and the common effective ablation area for the left and right coronary sinuses.

The representative examples of successful ablation of PVCs/VT originating from the left coronary sinus, the right coronary sinus, and the under left coronary sinus are shown in Figures 1, 2 and 3. The common distribution area of the effective ablation target site in the left coronary sinus group and in the right coronary sinus group is shown in Figure 4. The 3 patients with frequent PVC occurrence (the average PVC count ≥ 10000 times /24 h) were verified as having no structural heart disease.

Figure 1 showed a successful ablation of a PVC originating from the left coronary sinus. (A) The ECG at admission showed that frequent PVC resulted in a complete left bundle branch block (CLBBB) shape with a right axis deviation, whereas the QRS complex presented an rS(s) type on leads I and V1 to V3 , which currently is r_V1_>r_V2_. The aVR and aVL presented a QS type, QS_aVL_>QS_aVR_, whereas leads II, III, aVF, and V4 to V6 presented a great R wave, whereby the precordial lead transitional region was on lead V3. (B) The starting point of the local V wave, which was mapped in the left coronary sinus of the LVOT, was ahead of the QRS wave of the PVC in the surface ECG for 41 ms. The fracture-like potential of the peak could be seen at the starting sector of the V wave, whereas a small A and big V waves could also be seen as part of the target site. (C) X-ray images of the target site showed the ablation catheter in the left coronary sinus of LVOT, which was about 1.5 cm from the left main coronary artery ostium. (D) This point was considered as the target site, and ablation was performed at 55°C. The PVC disappeared after ablation for about 5.8 s, whereas consolidation of the ablation occurred for 180 s. The PVC subsequently disappeared, and no relapse occurred during the one-year follow-up.

Figure 2 showed a successful ablation of a PVC originating from the right coronary sinus. (Figure 2A) The ECG at admission showed frequent PVCs, of which the QRS wave presented the shape of CLBBB. R type was shown on leads II and III, aVF, and V3–V6. R_II_>R_III_, rS, and RS type were presented on leads V1 and V2. The height of the R wave and the depth of the S wave were 0.28 and 1.15, respectively. The wave duration of R and QRS was 0.50 and 0.57, respectively. For the R type on lead I, QS type appeared on leads aVR and aVL, QS_aVR_>QS_aVL_. The precordial lead transitional region was on lead V2, which indicated that PVC originated from LVOT. Thus, radio frequency catheter ablation operation was scheduled. The results demonstrated that the starting point of the local V wave, which was mapped in the right coronary sinus of LVOT (Figure 2D), was ahead of the QRS wave of the PVC in the surface ECG for 34 ms (Figure 2B). The QRS complex that originated from the right coronary sinus of the LVOT was the same as the spontaneous PVC on the 12 leads (Figure 2C). This point was considered as the target site, and ablation was performed at 55°C. The PVC disappeared after ablation for about 7 s, and consolidation of ablation occurred at 180 s. After the PVC disappeared, no relapse occurred during the follow-up at one and a half years (Figure 2).

Figure 3 showed a successful ablation of a PVC originating from the the under left coronary sinus. (A) The QRS complex of the PVC presented the shape of complete right bundle branch block (CRBBB). Her rs type appeared on lead I; QS type was on leads aVR and aVL. R type was on leads II and III, aVF, and V1 to V6 , with the R wave descending a notch on leads II, III, and aVF, which indicated idiopathic PVC originating from the under left coronary sinus of aorta. (B) The time of the earliest ventricular potential, which was mapped in the activation order in the under left coronary sinus of the aorta (left fibrous trigone) by the ablation catheter, was ahead of the spontaneous QRS complex of the PVC for 31 ms. Small A and big V waves were also observed. (C) Pacemaking mapped in this place was the same as that of random PVC in the 12 leads. (D) X-ray images at the target site showed that the ablation catheter was in the under left coronary sinus of the aorta (left fibrous trigone), which was considered as the target site and where ablation was performed at 55°C. The PVC disappeared when temperature reached 50°C for 3 s, and consolidation of ablation occurred at 180 s. After the PVC disappeared, no relapse occurred during the follow-up for two and half years (Figure 3).

The PVC that originated from the RVOT on leads V1 to V2 were taller and wider. The ratio of the R wave duration was ≥50%. The ratio of the R wave amplitude was ≥30%, and the precordial lead transition was between leads V1 and V2. Whether the index agreed with the origin, the LVOT was determined according to the report by Ouyang et al. [1]. Close observation of the sinus rhythm showed that R/S on lead V1 was equal to1, and the precordial lead transition was on lead V1. Transitional index = 1–1.5 = −0.5, which conformed to the origin of RVOT. Finally, a successful catheter ablation of PVCs was performed in the upper interval of the RVOT (Figure 4A1). The PVC on leads V1 to V6 that originated from the under left coronary sinus had a single R wave characteristic with a notch on the descending branch on inferior wall leads (Figure 4A4) (PVC that originated from the right coronary sinus had r or m type on lead I, and the precordial transition followed between leads V1 and V2, Figure 4A2). The PVC that originated from the left coronary sinus had rs type (r<s) on lead I, and the precordial transition emerged prior to the right coronary sinus (between leads V1 and V2, Figure 4A3). The effective ablation area in the left coronary sinus is usually located on the aortic sinus wall in front of the left main coronary artery, whereas that in the right coronary sinus is usually located on the aortic sinus wall in front of the right coronary sinus or right coronary valve (Figure 4B).

## Discussion

### ECG characteristics of the PVC that originated from LVOT

LVOT refers to the inner area of the aortic sinus and the under area of the aortic valve. The area under the aortic valve, which extends from the anterior lower lobe margin of the left atrioventricular valve to the aortic valve, has a length of about 10 mm. Some scholars consider the mechanism of PVC/VT that originate from this area as recurrently triggered by delayed after-depolarization [4-6].

The RVOT and LVOT are anatomically adjacent to each other. PVC/VT from the outflow tract consistently presented tall R waves in leads II, III, and aVF, unlike in other leads. Generally, the dominant wave of the QRS complex of PVC/VT from RVOT is in a downward direction and presents an rS type. The transition of the precordial leads was in lead V3 or behind lead V3. Conversely, the dominant wave of the QRS complex from the LVOT in lead V1 was in a downward or upward direction and presented rS, RS, or Rs type, whereas that in lead V2 was in an upward direction and presented an rs or Rs(r/s > 1) type, and then the transition area in the precordial leads was in or ahead of lead V2 [7]. Some patients presented r_V1_ > r_V2_, and the transition area in the precordial leads was between leads V2 and V3. In this circumstance, an observation can be derived from the time limit ratio of the R wave in leads V1 and V2 (time limit of R wave in lead V1 or V2/time limit of QRS wave) and from the amplitude ratio of the R wave (R wave amplitude in lead V1 or V2/ S wave amplitude). If the time limit ratio of the R wave is >50% and its amplitude rationis >30%, PVC originates from the left ventricle, and conversely, if the time limit ratio is <50% and the amplitude is <30%, the PVC originates from the right ventricle [1]. The ECG variation can be explained by the illustration in Figure 4B. PVCs/VT that originates from the LVOT (aortic sinus) manifestes as a taller and longer r(R) wave than that which originates from the RVOT, suggesting that forward depolarization of the LVOT myocardial was thicker and had longer depolarization time than that of PVC/VT from the RVOT. Although the diagnostic standard stated above had relatively higher sensitivity and specificity for differentiating PVC/VT from the LVOT and that from the RVOT, conditions can be easily misdiagnosed, such as thoracic deformity and heart transposition, or the position of the ball aspirator for precordial leads can be slightly shifted during ECG examination. When judging the origin of PVCs/VTs, researchers should examine the PVC/VT and the configuration of the QRS in precordial leads during sinus rhythms. Betensky et al. [8] reported that if the precordial lead transition of VT emerges after that of the sinus rhythm, the origin of the LVOT can be completely excluded. However, if the two elements are equal or the former emerges earlier, the transition ratio in lead V2 should be calculated by computing the percentage R-wave during PVC/VT (R/R+S) divided by the percentage R-wave in sinus rhythm (R/R+S). If the ratio is ≥0.6, 95% sensitivity and 100% specificity for predicting VT that originates from LVOT are manifested. Naoki Yoshida et al. [2] observed that the current criterion of judgment for the value of prediction in patients with heart transposition is limited. They developed the formula for the transitional area index, whereby the value of the lead is provided by the transitional area in the lead. In addition, the transitional area index denotes the difference between the transitional area during sinus rhythm and that during VT. The transitional area index of >0 had 88% sensitivity of prediction and 82% specificity for those from the LVOT.

In the present study, PVC/VT that originated from LVOT was investigated in 52 cases that underwent electrophysiological mapping and/or ablation. According to the origin of its effective target site or the first activation site, the patients in this study were divided into three groups: left coronary sinus, under left coronary sinus, and right coronary sinus group. The feature of the 12-lead surface ECG that originated from different sites of the LVOT was studied, whereas PVC/VT that originated from the RVOT in 104 cases, as confirmed by radio-frequency ablation during the same period of hospitalization, was analyzed and compared. With <V3 transition of precordial leads and >0 transitional index of precordial leads as diagnostic indexes for PVC/VT that originated from LVOT, we measured sensitivity as 94.23%, specificity as 91.35%, positive predictive value as 84.48%, and negative predictive value as 96.94%. Analysis of different subgroups of LVOT showed that most patients in the right coronary sinus group mainly presented r or m type on the positive waves in lead I (72.73%), which were significantly more than the 12.50% of the left coronary sinus group and none in the under left coronary sinus group (p < 0.01, both). However, accounting for 81.25% and 66.67%, respectively, the other two subgroups mainly presented rs or rS type, which were remarkably more than the 9.09% of the former subgroup (p < 0.01, both). Although all patients in the three groups presented R type in leads II, III, aVF, and V4 to V6, QS type was evident in the aVR and aVL leads. Differences were observed in the distribution of height of the R wave and depth of the QS wave. All patients in the right coronary sinus group exhibited R_II_>R_III_ and QS_aVR_>QS_aVL_. By contrast, the other two subgroups mainly presented R_III_>R_II_ and QS_aVL_>QS_aVR_ (both p < 0.01). Differences were also manifested in the shape of the QRS complex in the precordial leads among the three groups. Most patients in the under left coronary sinus group mainly presented R type in lead V1 on the positive wave (77.78%), while those in the right coronary sinus group (81.82%) and the left coronary sinus group (62.50%) mainly presented rS type on the negative wave. The transition of precordial leads for patients in the under left coronary sinus group was prior to lead V1, while those in the left and in the right coronary sinus groups were mainly in V2 to V3. Most patients had a notch on the descending portion of the Rwave in inferior leads in the under left coronary sinus group (88.89%), whereas no notch on the descending branch of the R wave in inferior leads in the other two groups was observed.

**Figure 5:**
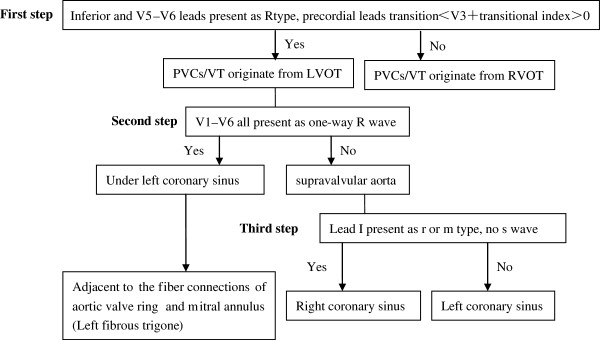
Differential procedure of PVC/VT in surface ECG originating from RVOT and LVOT.

PVC/VT from the LVOT can take place in the left, under left, right, and non coronary sinus groups. Their occurrence was clinically most common in the left coronary sinus group, followed by the right and the under left coronary sinus groups, whereas they were rarely seen in the no coronary sinus group. Clinically, patients who presented with the R type in the inferior and V5 to V6 leads and <V3 transition of precordial leads with >0 transitional index of precordial leads, should first be considered to have an origin in the LVOT. The differential procedure of PVC/VT originating from the RVOT, LVOT, and from other regions is shown in Figure 5.

### Ablation and methods for PVC originating from the LVOT

PVC/VT partly originated from the epicardium of the LVOT. Ablation was successfully performed in the structure on the vicinity of the LVOT epicardium. The most common application of epicardium for ablation was applied through aortic Valsava sinus (left coronary sinus). This site is in the anatomic vicinity of the epicardium of the ventricular upper septal myocardium, which actually originates from the epicardium of the septal myocardium. When PVCs/VT are ablated in the left coronary sinus, the ablation catheter should be carefully operated to avoid its entrance into the left coronary. Coronary arteriography must be routinely performed. If necessary, an angiography catheter should be utilized as an indicator to avoid damaging the coronary during ablation. Alteration of the intracardiac electrogram should also be observed carefully through the ablation catheter and by repeated intermittent X-ray exposure when the ablation is performed in the left coronary sinus. If the ablation catheter shifts, ablation should be discontinued immediately. In the present work, the ablation method for PVCs/VT in the right and in the under left coronary sinus was similar to that in the left coronary sinus. For PVCs/VTs that originate from the left coronary sinus, the ablation target site should be determined based on “activation sequence mapping.” This kind of pacing mostly cannot activate the ventricle. However, for PVCs/VTs that originate from the right and the under left coronary sinuses, the target site should be based on the integrity of the “activation sequence” and “pace mapping” because pace mapping can be worked out for most patients. The effective ablation target site is usually under the front of the ostium of the left main coronary artery in patients with the left coronary sinus as the origin. Hence, coronary angiography should be performed after ablation to confirm post-ablation patency of the coronary artery. Furthermore, coronary angiography should be performed subsequently after ablation to determine the condition of the blood supplement. In contrast, the effective ablation target site of patients with the right coronary sinus as the origin is usually on the aortic sinus wall before the right coronary valve and not from the ostium of the right coronary. However, some patients can possibly suffer from an abnormal ostium of the right coronary. Therefore, coronary angiography should be conducted before ablation to ensure the link of the location between the target site and the coronary. The discussed mapping methods most importantly consider the origin of the PVC/VT. Further, the occurrence feature of PVCs/VTs during operation can affect the selected mapping method. For patients with more PVCs during operation, the combination of “activation sequence mapping” and “pace mapping” results in a more rapid and accurate method in finding the target site. “Activation sequence mapping” is firstly selected for primary confirmation of the first areas of ventricular activation. Careful mapping on the location is then performed through “pace mapping.” For patients with less PVC during operation, the common way to locate an ideal target site is to use “pace mapping” in the original area predicted by surface ECG. Ablation is difficult during frequent occurrence of PVCs after the location is mapped by electrophysiology examination. Ablation should be discontinued if the PVC/VT is not terminated within 10 s during discharge. Otherwise, mapping should be performed again.

In addition, PVCs/VTs that are less than 0.5 cm away from the left main coronary artery or ostium of the main coronary artery should not be ablated as the tunica intima in coronary arteries can be injured by ablation, which would result in acute embolism formation and acute myocardial infarction [9,10].

## Conclusions

PVC that originates from LVOT is not uncommon. Most patients can safely and effectively apply mapping and ablation through routine mapping and catheter ablation with general temperature control through the aortic reversal path. Some differences in the 12-lead ECG of the LVOT with different origins are observed. Therefore, familiarity with these features is essential to determine initially possible effective target sites, to shorten the operation time, and to reduce X-ray exposure prior to operation.

## Competing interests

The authors declare that they have no competing interests.

## Authors’ contributions

LJF and GB designed the entire study, LJF, GB, JKT, LJ, LYC and YRP performed the experiments, LJF, GB and YHG wrote the paper. All authors read and approved the final manuscript.

## Pre-publication history

The pre-publication history for this paper can be accessed here:

http://www.biomedcentral.com/1471-2261/12/112/prepub
